# Association between variants of the autophagy related gene ATG16L1 in inflammatory bowel diseases and clinical statues 

**Published:** 2019

**Authors:** Shaghayegh Baradaran Ghavami, Fateme Kabiri, Mahyar Nourian, Hedieh Balaii, Shabnam Shahrokh, Vahid Chaleshi, Ghazal Sherkat, Farzaneh Shalileh, Hamid Asadzadeh Aghdaei

**Affiliations:** 1 *Gastroenterology and Liver Diseases Research Center, Research Institute for Gastroenterology and Liver Diseases, Shahid Beheshti University of Medical Sciences, Tehran, Iran. *; 2 *Basic and Molecular Epidemiology of Gastrointestinal Disorders Research Center, Research Institute for Gastroenterology and Liver Diseases, Shahid Beheshti University of Medical Sciences, Tehran, Iran *; 3 *Student Research Committee, Islamic Azad University, Mashhad Branch, Mashhad, Iran*

**Keywords:** Autophagy, ATG16L1, Inflammatory bowel disease, Diseases status

## Abstract

**Aim:**

In the present study, two main variants of ATG16L1 gene, rs2241880 T300A and rs2241879 C/T, were evaluated in IBD patients as well as in remission and flareup phase across an Iranian population for the first time.

**Background:**

Inflammatory bowel disease (IBD) has found increasing global incidence and prevalence in recent years especially among pediatrics. ATG16L1 is the major gene that regulates autophagy pathway. The autophagy pathway also affects dysbiosis.

**Methods:**

Genomic DNA was isolated from peripheral blood samples following salting out extraction method. The genotypes of ATG16L1 polymorphisms rs2241880 T300A and rs2241879 C/T were determined using polymerase chain reaction-restriction fragment length polymorphism (PCR-RFLP) method.

**Results:**

In this case control study, a total of 101 IBD patients (75 ulcerative colitis (UC) and 26 Crohn’s disease (CD)) and 99 healthy controls were evaluated. In the present study, a significant association was found between rs2241879 single nucleotide polymorphism on ATG16L1 gene and increased risk of IBD among an Iranian population (P=0.01). There was no statistically significant relationship between rs2241880 and IBD risk (P= 0.42). The effect on these two variants was investigated in relapse and flareup phase which was not significant either, but in CD, rs2241879 and rs2241880 were difference in the relapse phase.

**Conclusion:**

The results showed that ATG16L1 gene rs2241879 has a significant relationship with increased risk of IBD among an Iranian population. Individuals with C allele showed a significant relationship with 1.68-fold increased risk of IBD (P=0.01; adjusted OR=1.68; 95% CI=1.13-2.50).

## Introduction

 Inflammatory bowel disease (IBD) which includes Crohn’s disease (CD) and ulcerative colitis (UC) is a multifactorial disease (1, 2) whose exact etiology is not fully clarified (3, 4). IBD diseases consist of two phases, flareup and remission; so symptoms will return over time and a person may wax and wane throughout their life (5). It has been shown in several studies that different factors such as genetic, environmental conditions, starvation, genotoxic stress, and gut microbiota are involved in IBD (3, 6, 7). In a genome-wide association study (GWAS) in Europe, different genes such as NOD2/CARD15, ICOSLG, IRGM, IL-23, and ATG16L1 were identified (8-10). Among the genetic loci studied in IBD, those genes that play a role in the maintenance of the bacterial balance (homeostasis) and innate immune regulation such as autophagy could be more important than other genes (11, 12). The ATG16L1 and IRGM genes as known essential genes in autophagy and some other genes including NOD2/CARD15 in antigen processing play a critical role in gut microbiota homeostasis (9, 13). The autophagy-related 16-like 1 molecule (ATG16L1) which is encoded by the ATG16L1 gene (2q37) is a key component of a large protein complex crucial for autophagosome biogenesis.

Autophagy is a homeostatic process in a cell which is involved in damaged organelles and development as well as differentiation of cells (14, 15). It is also involved in regulating the secretion of the key pro-inflammatory cytokine IL-1 of the macrophages which controls host immunity defense (16) as well as some situations that lead to cellular stress such as starvation, genotoxic stress, and infection active autophagy pathway (15, 17). The special autophagy pathway selectively involves bacteria, virus, and other non-host entities called Xenophagy (10, 18). In this pathway, ATG16L1 encodes a small coiled-coil protein which interacts with ATG5 and ATG12 to form a 350 kDa multimeric complex which plays an important role in autophagy. Autophagosom complex plays an essential role in eliminating the pathogen (18). ATG16L1 is also found in the colon, small bowel, intestinal epithelial cells, leukocytes, and spleen (19). There are nine variants of the ATG16L1 gene, but two of these variants, rs2241880T300A and rs2241879 C/T have shown a significant association with CD (13, 15, 19). Lakatos et al. (20) showed that ATG16L1 gene rs2241880 T300A is a susceptibility locus for CD in Hungarian patients (P= 0.03, 95% CI=1.04-3.04). It has also been observed in Moroccan individuals who carry mutant allele of ATG16L1 gene rs2241880 T300A with a protective effect against UC (21). Also, rs2241879 C/T shows a significant relationship with CD in the German population (P=0.00)(22). Given that many studies have shown dysbiosis plays a critical role in IBD pathogenesis (23, 24), related genes that affect the microbiota hemostasis have an important position in IBD studies such as ATG16L1gene and its variants. The results of ATG16L1 polymorphism have been controversial in IBD prevalence in different continents (25, 26). Also, there are a few studies of various effective factors on IBD status (remission and flareup phase)(14, 27). The aim of the present study is to investigate the ATG16L1 gene rs2241880 and rs2241879 relationship with IBD and two phases of diseases amongst an Iranian population in Asia. 

## Methods

**Study population and data collection**

The study is a case-control study on 101 patients with IBD (75 UC and 26 CD) with 72 flareup and 29 remission status and 99 healthy controls referring to Taleghani Hospital, Tehran, Iran. Those with positive colonoscopy and pathologic findings for IBD (CD and UC) were included in the patient group and controls were checked for inflammatory diseases and malignancy. The study was approved by the ethics committee of the Research Institute for Gastroenterology and Liver Diseases, Shahid Beheshti University of Medical Sciences, Tehran, Iran. 

**Genotyping**

**Figure 1:**
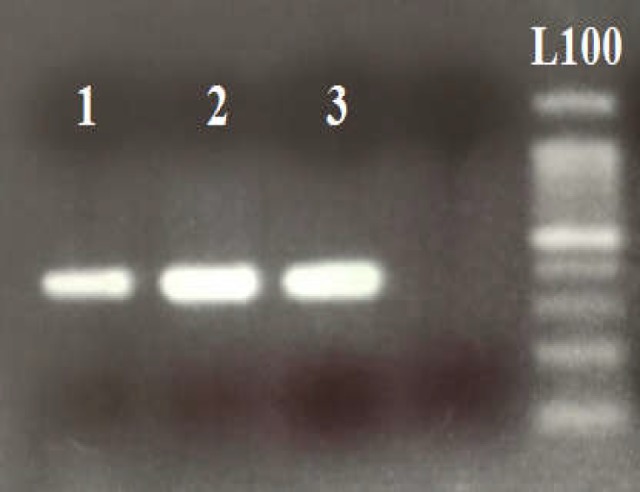
Amplification PCR products rs2241880, 386bp

Initially, 5 mL of peripheral blood was collected in EDTA-containing tubes and genomic DNA was extracted following salting out method (28). Genotyping of ATG16L1 rs2241880 was performed based on polymerase chain reaction-restriction fragment length polymorphism (PCR-RFLP). The 386-base pair PCR product was generated in 25 μL final mix containing 2.5 μL of 10X PCR buffer, 100 ng DNA template, 0.5 pM of each primer as shown in Table 1, 1 mM MgCl_2_, 0.5 μM of each dNTP, and 0.5 unit of Taq DNA polymerase. The amplification protocol was carried out under the following conditions: a denaturation step for 5 min at 94°C followed by 35 cycles of a denaturation at 94°C or 45 s, annealing at 63°C for 35 s, extension at 72°C for 45 s. A final extension was performed for 10 min at 72°C. DNA bands were detected using Ethidium bromide staining after being electrophoresed on 1% agarose gel (Roche, Germany) (Figure 1). 

**Table 1:** Designed primers and limited enzyme for desired components and the size PCR products and enzymatic digestion

SNP Reference ID	Primer sequence	Location (Base change)	PCR Product Size (bp)	Restriction Enzyme	RFLP fragments size (bp)
ATG16L1 rs2241880	F: 5'- AGGCTCTGTCACCATATCAAG -3' R: 5'- ACAGGTTAGTGTGCAGGAGA -3'	T/C	386bp	Bful1	C: 386 T: 266+120
ATG16L1 rs2241879	F: 5'- TGGAGTCCTTTCTAACAATTTG -3' R: 5'- CTGGCAACTCACTCTAAACT -3'	C/A	563bp	Tsp45I	A: 563 C: 265+222

**Figure 2:**
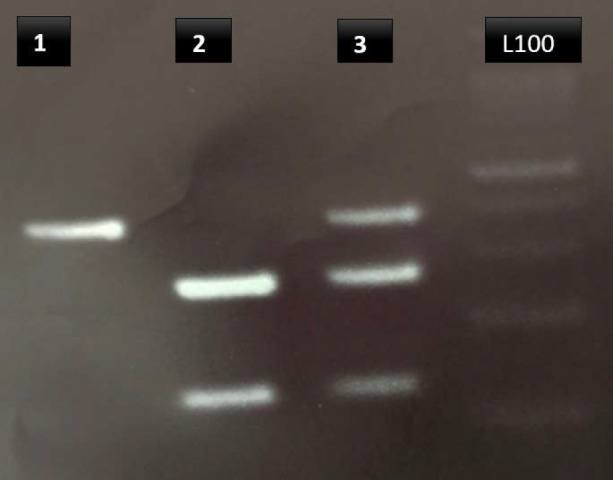
Digestion RFLP rs2241880 withe BfuI Enzyme: 1= CC, 386bp - 2= TT, 266bp-120bp - 3= CT, 386bp- 266bp-120bp

The PCR products were digested with 0.2 unit *BfuI *(Thermo scientific) endonuclease overnight at 37°C (Table 1). The digested PCR products were analyzed using electrophoresis on 3% agarose gel (Roche, Germany) and staining by Ethidium bromide. The results of digestion are shown in Figure 2.

**Figure 3:**
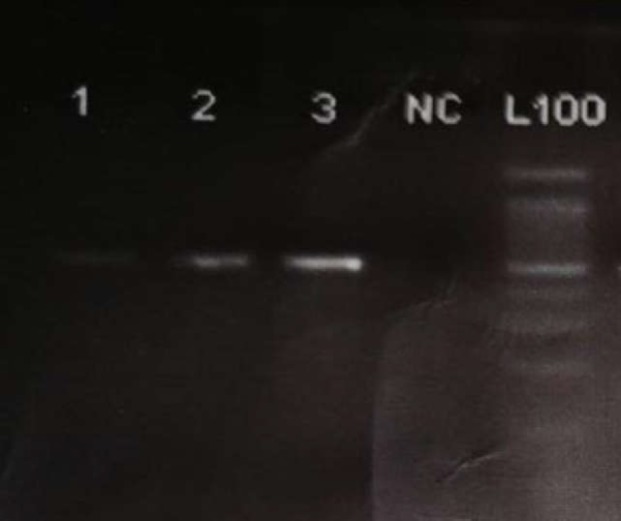
Amplification PCR products rs2241879, 563bp

The rs2241879 SNP was also detected using PCR-RFLP method. The 563 bp PCR product was amplified in 25 μL cocktail containing 100 ng genomic DNA, 2.5 μL of 10X PCR buffer (10 mM Trich-choloride, 50 mM chloride potassium 0.1%, Tritiun X-100) (Genefanavaran, Iran), 75 μL MgCl_2 _(Genefanavaran, Iran), 0.5 μL dNTP (Genefanavaran, Iran), 1 pM of each primer (Table 1), 0.25 units of *Taq* DNA polymerase (Genefanavaran, Iran) under the following conditions: a denaturation step for 5 min at 95°C followed by 35 cycles of a denaturation for 45 s at 94°C, annealing for 35 s at 55.8°C, extension for 45 s at 72°C and a final extension for 10 min at 72°C in a thermocycler (Eppendorf, Germany). DNA bands were detected using agarose gel electrophoresis on 1% agarose gel, followed by Ethidium bromide staining (Roche, Germany) (Figure3). 

**Figure 4:**
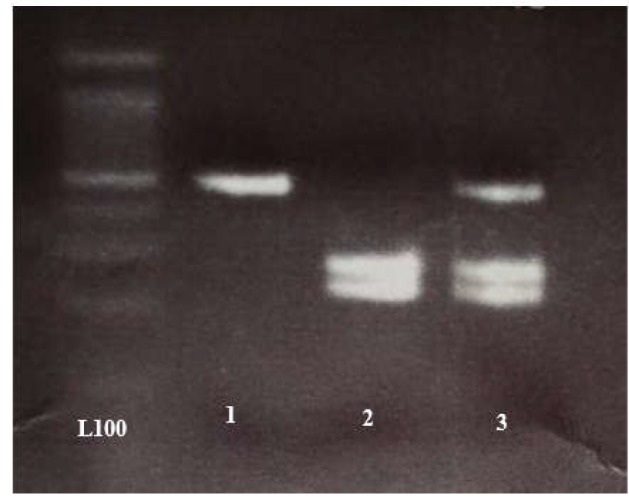
Digestion RFLP rs2241879 withe Tsp45I Enzyme: 1= AA, 563bp - 2= CC, 265bp- 222bp - 3= AC, 563bp- 265bp- 222bp

The PCR products were digested with 0.1 units of *Tsp45I *(Thermo scientific made in Eu Lithuania) endonuclease overnight at 37°C. The digested PCR products were analyzed using electrophoresis on 3% agarose gel (Roche, Germany) (Table 1) and Ethidium bromide staining. The digested fragments are displayed in Figure 4.

**Sequencing**

Sequencing method was used for 10% of the PCR products to confirm the RFLP procedure using an ABI PRISM 3130xL Genetic analyzer (Applied Biosystems®, Invitrogen Life Technologies, USA). 

**Statistical analysis**

Statistical analysis was performed using the Statistical Package for Social Sciences software (SPSS) version 24 with P≤0.05 set as the significance. Logistic regression analysis was applied to estimate odds ratios (OR) and 95% confidence intervals (CI). The Hardy-Weinberg gene equilibrium was examined using Chi-square test. Age, sex, and recruitment source (two subject groups) were adjusted to exclude any potential confounder. 

## Results

The study population consisted of 26 patients suffering from CD with a mean age of 32.8813.6 years (12 males and 14 females) and 75 patients with UC with a mean age of 36.2411.75 (46 males and 29 females); the control group’s mean age was 35.2713.6 (32 males and 67 females) (Table 2). The characteristics of case and control individuals enrolled in the present study are shown in Table 2. The genotype and allele frequencies of ATG16L1 rs2241880 T300A and rs2241879 C/T among the patients and control subjects are shown in Table 3. In the present study, a significant relationship was found between rs2241879 and risk of IBD in the Iranian population. CC genotype showed a protective effect on the risk of IBD (P=0.01; adjusted OR=1.68; 95% CI=1.135-2.506). Also, adjustment was done for confounding factors including BMI, age, and smoking status. A significant relationship was also found between A allele of rs2241879 and increased risk of IBD (P<0.01; adjusted OR=1.68; 95% CI=1.13-2.50). Patients with A allele showed a significant relationship with 1.68-fold increased risk of IBD as compared to the control. The other single nucleotide polymorphism (SNP) of ATG16L1 gene, rs2241880 T300A did not show any significant relationship with CD and UC (P=0.42; adjusted OR=0.85; 95% CI=0.57-1.26). The variants of ATG16L1 gene, rs2241880 and rs2241879, of patients with CD and UC were evaluated in remission and flareup status for the first time where slight differences were observed between Chrons patients in the flareup phase (P=0.06 and P= 0.07, respectively). Allele C could increase the risk of relapse. There are more details in Table 4. Also, rs2241879 showed that IBD patients with AA genotype were in the remission phase more than with any other genotype; in other words, the A allele may play a protective role in relapse.

## Discussion

Various studies shown that a major factor in IBD etiology is gut microbiota dysbiosis (29). So, different pathways and host genetic variants are implicated in the variability of the gut microbiome among individuals. Autophagy can control intracellular bacteria whose malfunction leads to overgrowth of a group of bacterial and dysbiosis (30, 31). Also, in this pathway, the ATG16L1 and IRGM are important genes that can be effective on the formation of autophagosoms’ protein. Defects in this protein complex results in abnormal bacterial killing and defective antigen presentation (18, 32). Autophagy by stimulating serine-threonine kinase 2 (RIPK-2) and activating cascade of TLRs pathway on dendritic cells helps regulate innate and adaptive immune system (30). The role of ATG16L1-deficient macrophages has been shown to increase inflammatory cytokines, IL-1, and IL-8, induced by gram-negative bacteria such as *Salmonella *species*,* and adherent-invasive *Escherichia coli *(AIEC)(31, 33). Several studies have shown that SNPs, the most common variants in genome, are able to modify and increase or decrease the risk of different diseases such as autoimmune diseases, cancer, and infections (34, 35) and are considered as biomarkers related to IBD susceptibility predicting its relapse (36). Also, genetic factors account for 20% to 40% of inter-individual differences in metabolism and response (37), even investigations on the role of SNPs as biomarkers for prediction of treatment response. Different studies have considered that several factors such as environment, lifestyle, and the composition of microbiota that it is effect on genetic variation of papulation, So spacious studies have recommended that it investigated genetic variation in different countries is important(2). In this study, the relationship between rs2241880 T300A and rs2241879 C/T polymorphisms in the ATG16L1 gene and IBD susceptibility in an Iranian population were explored. It was postulated that alterations on these regions might significantly modify the function of autophagy pathway and in addition to several studies have illustrated that the increased risk of IBD diseases (15). In the present study, a significant relationship was found between rs2241879 of ATG16L1 gene and risk of IBD in Iranian population. CC genotype has a protective effect against IBD. Patients with Allele A of rs2241879 show significant relationship with 1.687-fold increased risk of IBD when compared with the control, which is consistent with previous findings. A study (22) showed the strongest relationship between rs2241879 with CD in German population (P=3.610^-6^; OR=0.74; 95% CI=0.65-0.84). Also, they showed a CD-protective effect for the minor allele. In another study in an Italian population, it was shown that ATG16L1 rs2241879 variant revealed a relationship with smoking (P=0.03) and G allele shows a protective role to CD (P=0.006; OR=0.03; 95% CI=0.002-0.45). An association of ATG16L1 rs2241879 with lack of extra-intestinal manifestations was also observed (P=0.006)(8). It was revealed that rs2241879 ATG16L1 may protect inflammation in several organs by modulating the generation of a self-tolerant T-cell through autophagy(8). No remarkable relationship was found between rs2241880 SNP of ATG16L1 gene in IBD patients and the healthy control group in Iranian population. Our results are similar to those of Asian populations, but on the contrary to other findings in the continents (25, 26, 38). In a German population, the strongest relationships were found for the coding SNP rs2241880 (T300A) (P=3.710^-6^; OR=0.74; 95% CI=0.65-0.84)(39). Lakatos et al.(20) revealed the relationship between IL-23 rs11209026, ATG16L1 rs2241880, and CD and no difference was found between patients with UC and either control or CD. The Hungarian patients with ATG16L1 300A/A had more risk of CD restricted to the colon. Lauriola (8) showed that NOD2/CARD15 and ATG16L1 are not the major contributors to CD susceptibility in an Italian population. Note that the different geographical origin could play a limited role in pathogenesis of CD. In a cohort study in UK, it was reported that carriers of the mutant allele have 1.35 to 1.45-fold higher risk for CD, but no relationship was found in UC (8). Recently, some studies have shown that ATG16L1 and NOD2 variation can be considered as integrated factors on response to biological treatment and increased risk of relapse (36). In our study, the relationship between ATG16L1 rs2241880 and rs2241879 was evaluated with flareup and remission status where no relationship was found, but both SNPs especially in CD patients had a weak link with flareup among a Japanese population (36). 

**Table 2:** Demographic characteristics of the IBD study population

Controls(N= 99 )	UC(N= 75 )	CD(N=26 )	variable
35.2713.6	36.2411.75	32.8813.6	age (mean±SD)
25.654.75	23.794.59	19.526.61	BMI^a^
			Diseases Statuts
-	54 (72.0)	18 (69.2)	Flare up (%)
-	21 (28.0)	8 (30.8 )	Remission (%)
			Gender, n (%)^b^
32(32.3)	46(61.3)	12(46.2)	Female
67(67.7)	29(38.7)	14(53.8)	Man
			Somling, n (%)^b^
12(12.0)	7(9.3)	3(11.5)	Smoker
88(88.0)	68(90.7)	23(88.5)	Non-smoking

a According to the Student’s t-test results; b According to the chi square test results.

**Table 3:** Allele and genotype distribution of the studied rs2241880 and rs2241879 among ulcerative colitis, Crohn's disease and healthy control subjects

SNPs	UC	CD	Control	P-Value	Adjusted* OR (95%CI)
ATG16L1 rs2241880					
CC (%)	30(40.0)	9(34.6)	30(43.5)	Ref	1.00 (Reference)
CT (%)	35(46.7)	12(46.2)	56(54.4)	0.79	1.14(0.42-3.08)
TT (%)	10(13.3)	5(12.2)	14(48.3)	0.44	1.66(0.45-6.15)
Allele					
C (Risk frequency) (%)	95(63.8)	30(56.6)	116(58.0)	Ref	1.00 (Reference)
T (%)	54(36.2)	23(43.4)	84(42.0)	0.42	1.17(0.78-1.70)
ATG16L1 rs2241879					
AA (%)	32(42.7)	8(30.8)	26(39.4)	Ref	1.00 (Reference)
CA (%)	31(41.3)	12(46.2)	44(50.6)	0.17	0.63(0.33-1.21)
CC (%)	12(16.0)	6(23.1)	30(62.5)	0.01	0.39(0.18-0.83)
Allele					
A (Risk frequency) (%)	95(63.8)	28(52.8)	96(48.0)	Ref	1.00 (Reference)
C (%)	54(36.2)	25(47.2)	104(52.0)	0.01	1.68(1.13-2.5)

* Adjusted for Age and gender as confounder variables

Different studies have found that ATG16L1 and NOD2 genes have an important role in susceptibility to IBD diseases. Our study across an Iranian population for the first time showed that this gene can affect the relapse during the period of treatment in addition to the increased risk of IBD. The important note of this gene in different variations, possibly in various societies, is its impact on increasing the risk of IBD. In this study, we suggest the need to consider the important variations of ATG16L1 and to study it in more populations as well as in different countries especially in Asia to achieve a general conclusion.
